# Radiological Screening Methods in Deceased Organ Donation: An Overview of Guidelines Worldwide

**DOI:** 10.3389/ti.2022.10289

**Published:** 2022-05-19

**Authors:** K. A. Chotkan, J. W. Mensink, R. A. Pol, N. P. Van Der Kaaij, L. F. M. Beenen, W. N. Nijboer, B. Schaefer, I. P. J. Alwayn, A. E. Braat

**Affiliations:** ^1^ Department of Surgery, Division of Transplantation, Leiden University Medical Center, Leiden, Netherlands; ^2^ Department of Organ and Tissue Donation, Dutch Transplant Foundation, Leiden, Netherlands; ^3^ Department of Surgery, Division of Transplantation, University Medical Center Groningen, Groningen, Netherlands; ^4^ Department of Cardiothoracic Surgery, University Medical Center Utrecht, Utrecht, Netherlands; ^5^ Department of Radiology, Amsterdam UMC, Amsterdam, Netherlands; ^6^ Transplant Center, Leiden University Medical Center, Leiden, Netherlands

**Keywords:** screening, transplantation, ethics, organ donation, organ procurement, imaging, guidelines, transplant ethics

## Abstract

Organ transplantation is performed worldwide, but policies regarding donor imaging are not uniform. An overview of the policies in different regions is missing. This study aims to investigate the various protocols worldwide on imaging in deceased organ donation. An online survey was created to determine the current policies. Competent authorities were approached to fill out the survey based on their current protocols. In total 32 of the 48 countries approached filled out the questionnaire (response rate 67%). In 16% of the countries no abdominal imaging is required prior to procurement. In 50%, abdominal ultrasound (US) is performed to screen the abdomen and in 19% an enhanced abdominal Computed Tomography (CT). In 15% of the countries both an unenhanced abdominal CT scan and abdominal US are performed. In 38% of the countries a chest radiographic (CXR) is performed to screen the thorax, in 28% only a chest CT, and in 34% both are performed. Policies regarding radiologic screening in deceased organ donors show a great variation between different countries. Consensus on which imaging method should be applied is missing. A uniform approach will contribute to quality and safety, justifying (inter)national exchange of organs.

## GRAPHICAL ABSTRACT

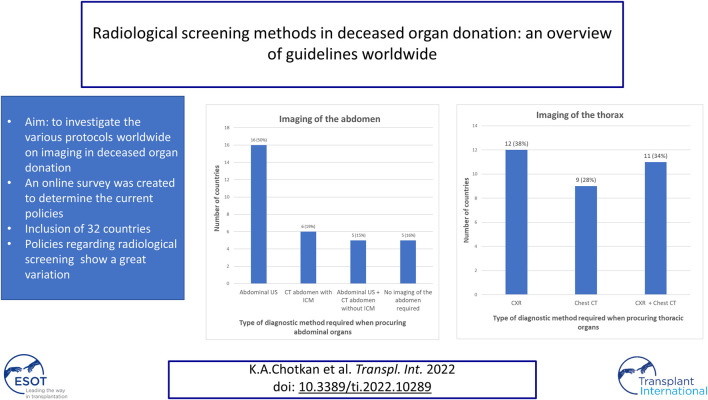

## Introduction

Organ transplantation is a lifesaving treatment for patients with end-stage organ failure but is not without risk for the recipient. The comprehensiveness and quality of donor assessment contribute to adequate risk management, applicable to individual and vulnerable recipients. Optimal donor assessment provides important information on organ quality and anatomy. Donor assessment includes interviews with relatives, assessment of the medical and social behavior history, full physical examination, laboratory tests, and complementary tests (in particular imaging) (1). In Netherlands (part of the Eurotransplant region), radiological screening in deceased organ donors consists of at least a chest radiography (CXR) and abdominal ultrasound (US). Various studies in the past have advocated for the inclusion of the use of chest and abdominal Computed Tomography (CT) scans to optimally prepare a donor and identify risk factors (2–4). Possible advantages of the use of CT scans are more accurate screening for malignancies and other significant diseases, mapping of aberrant (vascular) anatomy, enhanced assessment of organ quality, and improved size matching in liver and lung transplantation.

More detailed imaging may also have a downside; incidental findings on chest and (un)enhanced abdominal CT scans have a prevalence ranging from 40% to 75%. Of these, 3%–20% findings require additional investigations (5–8). This could possibly lead to more (invasive) diagnostic procedures with potential risks and could delay the procurement and allocation process. On the other hand, when being informed pre-operatively of these findings, biopsies can be obtained before procurement.

Also, to perform an enhanced CT scan, intravenous contrast medium (ICM) must be administered, which leads to exposure of donor kidneys to a potential nephrotoxic contrast medium. A recent publication of Magnus et al., containing a retrospective analysis of 709 kidney donors who received ICM, showed no difference in serum creatine levels in the donor, delayed graft function (DGF) or graft loss in the recipients compared to 685 kidney donors who did not receive ICM (9). This group only contained Donation of Brain death (DBD) donors and no Donation after Circulatory Death (DCD) donors. The DGF rate in DCD kidneys is known to be significantly higher compared to DBD kidneys (10). The added effect of ICM may therefore have an even higher (negative) impact on outcome by inducing acute kidney injury (AKI). Finally, transport to the radiology department of a critically ill patient adds additional risks.

Although organ transplantation is performed worldwide, policies regarding donor assessment and imaging are not uniform. An overview of the policies and underlying arguments in different regions of the world could provide valuable information for countries who are thinking about changing their policy. A uniform approach will contribute to quality and safety, justifying (inter)national exchange of organs.

This study therefore aims to provide an overview on the various protocols for radiological screening in deceased organ donation worldwide.

## Materials and Methods

To investigate whether an overview of the different policies in organ donor screening was available, a literature search of PubMed was performed, using Mesh terms; diagnostic imaging, tissue donors, tissue and organ procurement (Supplementary Appendix S1).

Additionally, an online survey was created in Survey Monkey to obtain country specific information (Supplementary Appendix S2). For information on countries with an active deceased organ donation program, and the annual number of (deceased) donors, the website International Registry in Organ Donation and Transplantation (IRODaT) was consulted (11). From 71 countries with a deceased organ donation program, transplant authorities were selected if they reported a total of at least 30 deceased donors per year (donation activity), based on the numbers of 2019, since 2020 is not representative due to the SARS-CoV-2 pandemic. This led to an inclusion of 48 countries. The value of a minimum of 30 deceased donors per year was chosen to include a large diversity of countries, including smaller countries, but to exclude countries which do not have deceased donation on a regular basis (and most likely do not have standardized guidelines for deceased organ donation). Contact information of these selected countries was obtained from Eurotransplant International, the Dutch Transplant Foundation and websites of the competent authorities of organ donation or donation professionals. Between May and July 2021, these contacts were approached by email to fill out the questionnaire.

To answer the question of whether imaging policies were associated with donor rate and donation activity, statistical analyses were performed using IBM SPSS Statistics for Windows (IBM Corp. Released 2017. Version 25.0. Armonk, NY). Shapiro-Wilk tests were used to assess the distribution of donor rate/donation activity between the imaging groups. To compare skewed numerical data the Kruskal Wallis test was used.

## Results

An overview of different guidelines regarding radiological screening in deceased organ donation was not found in PubMed. The Guide to the quality and safety of organs for transplantation from the council of Europe (1) has a specific chapter on donor imaging. In this chapter it is advised that at minimum, an up-to-date CXR and abdominal US should be included at the time of donation. Further radiological tests are advised to be performed when thorough donor evaluation is required, for example in patients with suspected malignancies or in donors in whom it is thought that appropriate intra-operative examination of the thoraco-abdominal cavities cannot be adequately carried out.

Thirty-two out of 48 countries on six continents responded to the questionnaire (response rate 67%). Table 1 gives an overview of all the diagnostic screening methods reported in the survey, including the number of deceased donors PMP (per million people) per country. Supplementary Datasheet 3 provides an overview of how many countries per region have been approached and the response rate per region. Three organizations did not give permission to publish their answers. Although these are not included in Table 1, their answers were analysed anonymously. Some countries replied that the guidelines were region dependent and do not apply to the whole country. This is also included in Table 1. Also, three respondents mentioned that guidelines describe the minimal requirements and that the accepting transplant centre could ask for additional examinations.

**TABLE 1:** Overview of the screenings method used in which country.

Country	Screening of the thorax when only thoracic organs are being procured	Screening of the abdomen when only thoracic organs are being procured	Screening of the thorax when only abdominal organs are being procured	Screening of the abdomen when only abdominal organs are being procured	Number of deceased donors PMP (per million people) in 2019	Guidelines used in the whole country
Australia/ New Zealand	Chest X-ray (for lung donors only if they meet certain criteria a chest CT is performed)	No Imaging performed of the abdomen	Chest X-ray	No Imaging performed of the abdomen	Australia: 20.10	Yes
					New Zealand 12.40	
Austria	Chest X-ray	Abdominal ultrasound	Chest X-ray	Abdominal ultrasound = minimal mandatory	20.30	Unknown
				In daily practice abdominal ultrasound and CT		
Belarus	Chest CT	Abdominal ultrasound	Chest X-ray	Abdominal ultrasound	26.20	Unknown
Belgium	Chest X-ray and chest CT	Abdominal ultrasound	Chest X-ray and chest CT	Abdominal ultrasound	27.20	Yes
Canada	Chest X-ray	None	Chest X-ray	None (Abdominal imaging is only advised in those with age >50, comorbid conditions, high BMI or clinical history of malignancy)	21.87	Yes (But every transplant region can ask for additional examinations)
Croatia	Chest X-ray	→ very rarely only thoracic organs, but if it happens, abdominal ultrasound	Chest X-ray	Abdominal ultrasound	31.20	Unknown
Czech Republic	Chest X-Ray and Chest CT (→ due to COVID)	Abdominal ultrasound	Chest X-Ray and chest CT	Abdominal ultrasound + CT abdomen without ICM	24.98	Yes
Ecuador	Chest X-ray and chest CT	Abdominal US	Chest X-ray	Abdominal ultrasound + CT abdomen without ICM	7.78	Unknown
Estonia	Chest X-Ray and chest CT	Abdominal ultrasound + CT abdomen without ICM	Chest X-Ray and chest CT	Abdominal ultrasound + CT abdomen without ICM	18.87	Yes
Finland	Chest CT	None	Chest X-ray and CT thorax	CT abdomen with ICM	25.51	Yes (only one transplantation centre in Finland)
France	Chest CT	CT abdomen with ICM	Chest CT	CT abdomen with ICM	33.25	Yes
Germany	Chest X-ray (if CT/MRT is done, it is always covering thorax and abdomen)	Abdominal ultrasound	Chest X-ray (if CT/MRT is done, it is always covering thorax and abdomen)	Abdominal ultrasound (CT/MRT whenever possible, ICM depends on the individual situation)	10.8	Yes
Greece	Chest CT	Abdominal Ultrasound	Chest X-ray	Abdominal ultrasound	5.0	No
Hungary	Chest X-ray and Chest CT	Abdominal ultrasound	Chest X-ray	Abdominal Ultrasound	18.11	Yes
Iran	Chest X-ray and Chest CT	Abdominal ultrasound	Chest X-ray	Abdominal ultrasound	14.34	Yes
Israel	Chest CT	CT abdomen with ICM	Chest CT	CT abdomen with ICM	10.43	Yes
Italy	Chest X-Ray and Chest CT	Abdominal ultrasound	Chest X-ray	Abdominal ultrasound	22.80	Yes
Japan	Chest X-ray	Abdominal Ultrasound	Chest X-ray	Abdominal ultrasound and CT abdomen without ICM	0.98	No
Netherlands	Chest X-ray	Abdominal ultrasound	Chest X-ray	Abdominal ultrasound	14.47	Yes
Norway	Chest X-ray and Chest CT	CT abdomen without ICM	Chest X-ray and chest CT	CT abdomen with ICM	18.18	Yes
Slovenia	Chest X-ray	Abdominal ultrasound	Chest X-ray and chest CT	Abdominal ultrasound	18.26	Yes
South Africa	Chest X-ray	No standard imaging of the abdomen required		No standard imaging of the abdomen required	1.29 (2016)	No
South Korea	Chest X-Ray and Chest CT	Abdominal ultrasound + CT abdomen without ICM	Chest X-Ray and CT thorax	Abdominal ultrasound + CT abdomen without ICM	8.68	Yes
Spain	Chest X-ray + Chest CT	Abdominal ultrasound	Chest X-ray	Abdominal ultrasound	49.61	Yes
Sweden	Chest CT	CT abdomen without ICM	Chest CT	CT abdomen with ICM	18.51	Yes
Switzerland	Chest X-ray + Chest CT (→ criteria defined by the lung expert group)	Abdominal ultrasound (→ when CT thorax is included, a CT abdomen is asked as well)	Chest X-ray	Abdominal ultrasound	19.30	Yes
Thailand	Chest X-ray	None	Chest X-ray	Abdominal Ultrasound (if indicated)	4.51	Yes
United Kingdom	Chest X-ray	No	Chest X-Ray	No	23.01	Yes
		Imaging performed of the abdomen		Imaging performed of the abdomen		
United States	Chest X-ray	Abdomen→ none	Chest X-ray	None	36.88	Yes (But every transplant region can ask for additional examinations)

Only the countries who gave permission to name their country were included in this table.

### Procurement of Abdominal Organs

**FIGURE 1:**
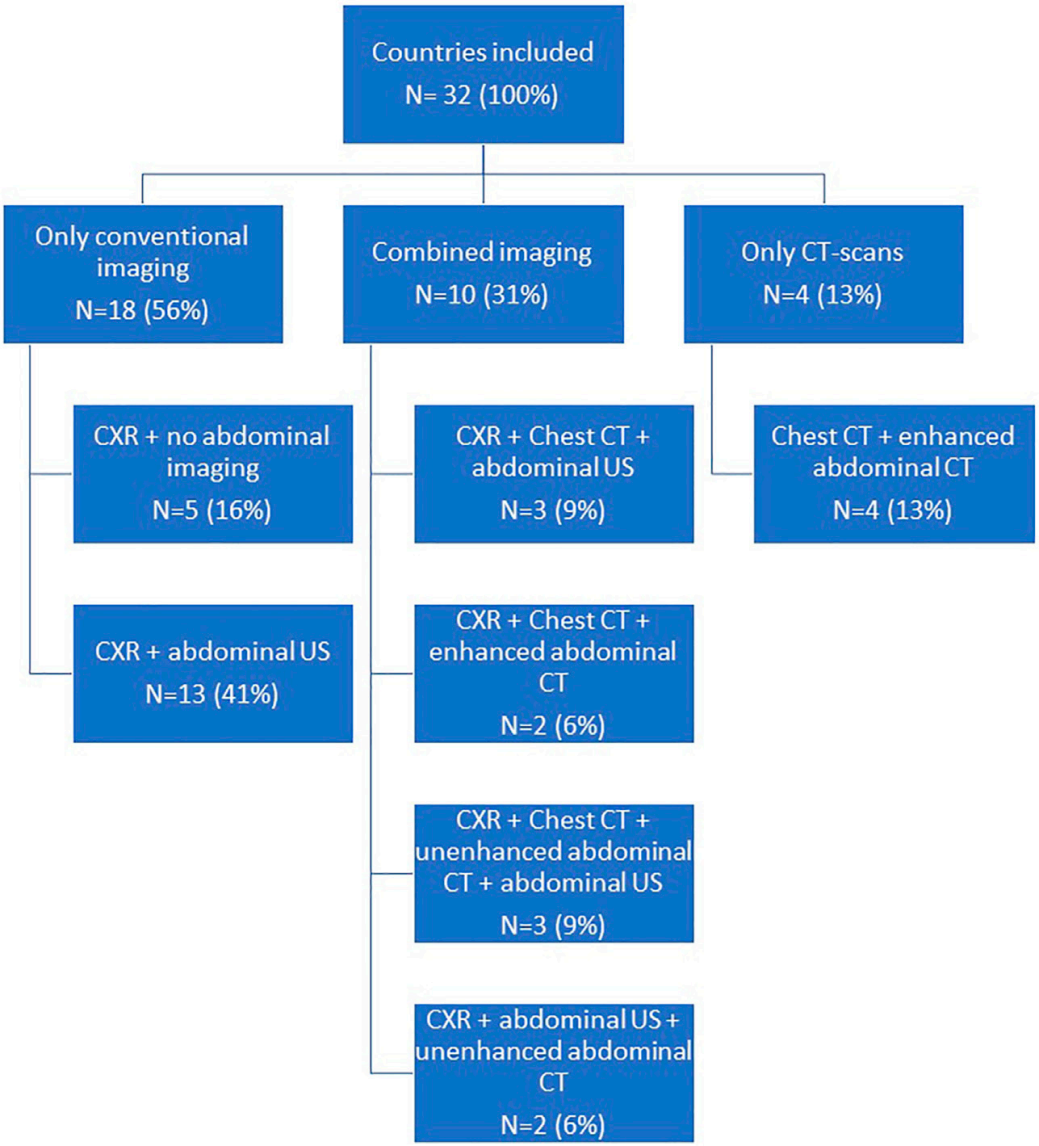
Flow chart on imaging performed when procuring abdominal organs.

**FIGURE 2:**
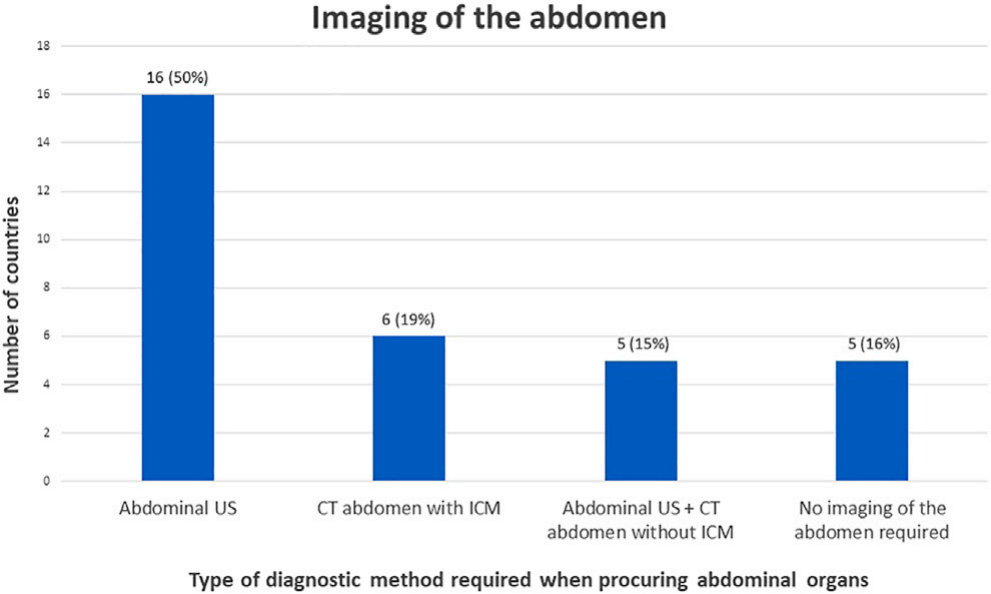
Graphical view of number of countries in which a certain policy is applied regarding imaging of the abdomen.

For the assessment of abdominal organ quality, CXR and abdominal US is considered the preferred screening method in 41% countries (Figures 1, 2). In 9% an abdominal US is performed in combination with a chest CT instead of a CXR. In 13% of the countries a chest and abdominal CT scan is part of the regular screening of deceased donors, in 6% next to these two imaging methods also a CXR is performed. In Finland, Norway, Sweden, France, and Israel an enhanced abdominal CT is made, excluding donors with existing or high risk for acute kidney injury (AKI). Unfortunately, the definition of what was considered a high-risk kidney donor was not further explained. In 15% of the countries an abdominal US as well as an unenhanced abdominal CT is performed. In 16% of the countries there are no minimal requirements regarding abdominal imaging prior to procurement and only a CXR is considered necessary.

### Procurement of Thoracic Organs

**FIGURE 3:**
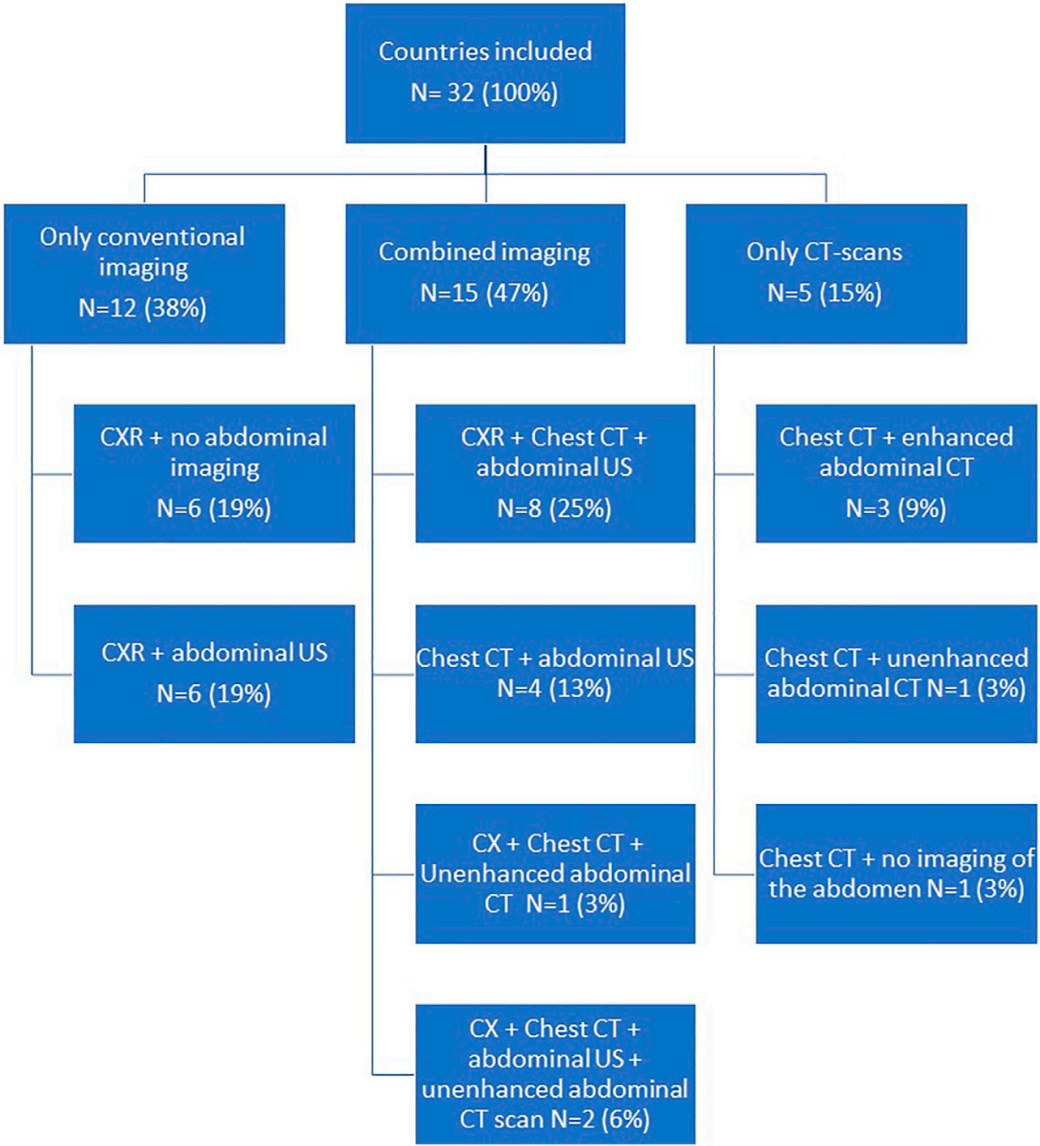
Flowchart on imaging performed when procuring thoracic organs.

**FIGURE 4:**
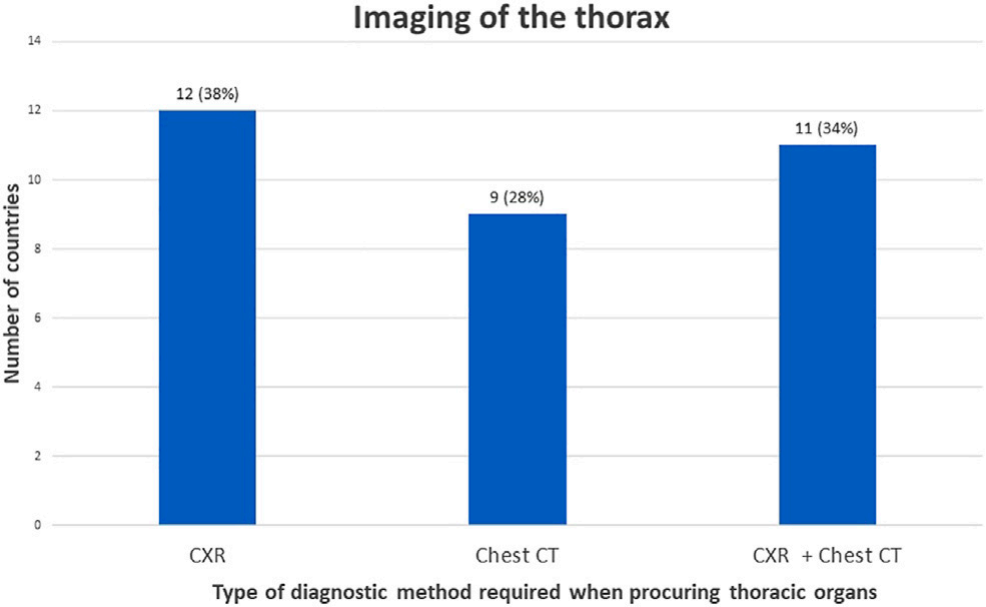
Graphical view of number of countries in which a certain policy is applied regarding imaging of the thorax.

To determine suitability of thoracic donor organs only a CXR is required in 19% of the countries, with no requirements of imaging of the abdomen. (Figures 3, 4). A CXR and abdominal US were considered the preferred screening method in 19% of the countries. In 25% a CXR, chest CT and abdominal US is performed. In 13% both a chest CT and abdominal US is carried out. In 9% of the countries chest CT and enhanced abdominal CT scan is performed. In 3% a CXR, chest CT and an unenhanced abdominal CT scan is made. A CXR, chest CT, an unenhanced abdominal CT scan plus abdominal US are performed in 6% of the countries. In 3% a chest CT and unenhanced abdominal CT scan was required, and another 3% required only a chest CT and no imaging of the abdomen.

### Summary of Preferences

Most countries (81% of the respondents) report that there are no objections against using CT scans in the screenings process of deceased donor organ donation. The reasons CT-scans are preferred are to facilitate the detection of malignancies (76% of the respondents were in favour of CT scans), and provide information about (aberrant) anatomy of the donor (68%). Sixty-four percent also reported CT scans have a value in providing information about organ quality, for example liver steatosis, renal atrophy, severe atherosclerosis, or pulmonary embolism.

Six respondents (16%) replied that there are objections for the routine use of CT scans in the screening process but addressed concerns regarding incidental findings that would unintentionally lead to donor rejection. Other objections were the logistic challenges associated with performing a routine donor CT, i.e., transporting the donor to the CT and increasing costs of the donation process.

If an abdominal CT scan is not part of the standard screening protocol, 76% of the respondents replied that the main reasons for performing an abdominal CT scan is for the purpose of trauma screening, or suspected anomalies detected on the conventional imaging (24%).

If a chest CT scan is not part of the standard screening protocol, 45% of the respondents replied that the main reason for performing a chest CT scan is also for the purpose of trauma screening or suspected anomalies on the conventional imaging (36%). Two respondents replied that reasons for making a chest CT scan was intended for screening for SARS-CoV-2.

Donor rate versus imaging policy was plotted, to investigate whether there is an association between imaging policies before procurement and donation rate (Supplementary Datasheet 4). No clear association was seen between these two using eyeball estimation. Using the Kruskal Wallis test, since the data was not normally distributed, no significant difference in donor rate between the different imaging policy groups was found (*p* = 0.37).

Donation activity (the total number of deceased donors per year) versus imaging policy was also plotted, to investigate whether there is an association between imaging policies before procurement and donation activity (Supplementary Datasheet 5). No clear association was seen between these two using eyeball estimation. Using the Kruskal Wallis test, since the data was not normally distributed, no significant difference in donation activity between the different imaging policy groups was found (*p* = 0.61).

## Discussion

This study shows a large difference between policies regarding diagnostic screenings methods in deceased organ donation in different transplant regions. The current literature lacks a consensus regarding imaging of deceased donors. No significant association between donor rate and imaging policy groups before procurement was found, nor a significant association between donation activity and imaging policy groups. The donor rate of the countries included ranged from 1 to 50 deceased donors PMP. The donation activity of the countries included ranged from 44 deceased donors per year to 11.870 deceased donors per year.

In the Eurotransplant International region (including eight European countries), the age of the donor population is increasing and with it also the comorbidity rate (12). Since this has impact on the incidence of malignancies and organ quality, an intensified assessment using radiological imaging has become increasingly important (13). Also the proportion of DCD donors has increased through the years, a donor pool historically known for its comorbidity and a rapid and mainly cold dissection, without proper perfusion feedback, in which prior knowledge of the anatomy significantly aids to the operative plan (14, 15).

In Finland, Norway, Sweden, France and Israel imaging is performed using chest and enhanced abdominal CT scan. On the contrary, Australia, the United Kingdom and South Africa do not require imaging of the abdomen before procurement of abdominal organs. In the United States and Canada there is no national policy on imaging of the abdomen, but the different Organ Procurement Organisations do have their own policies. In South Africa there is no screening of the abdomen because of costs and logistic challenges, but in Australia this is a well-considered choice because the procuring surgeon always performs an examination of the abdominal cavity and organs. The idea is that the added yield of abdominal imaging is low and could potentially extend the donor work up time (due to evaluation of any abnormalities). The United Kingdom stated that, in their opinion, performing an abdominal US has no additional value. Detection of malignancies depends on exploration of the abdomen by the procuring surgeon, an approach that might work for large tumors but is expected to have a low sensitivity and specificity for smaller of intraparenchymal lesions. With the shift in the donor population towards more older and extended criteria donors, we as professionals should start asking the question of whether it is time for a paradigm shift. Furthermore, it is interesting to note that English-speaking countries tend to avoid imaging prior to procurement, which could suggest there might be a cultural or historical reason for this.

There were conflicting ideas reported regarding the risk of administrating ICM to potential kidney donors. France, Israel, Sweden, and Norway (all four using enhanced abdominal CT scans) are only reluctant giving donors with a marginal kidney function ICM. But what is considered a marginal donor is often poorly reported or defined. Except for Israel, which uses a specific definition, in which donors with an increase in serum creatinine of more than or equal to 50% from baseline, a creatinine level of >150 μmol/L or a reduction in urine output to less than 0.5 ml/kg/h for more than 12 h despite adequate hydration, are excluded. (Of note; this is slightly different from the AKI classification of AKI stage 1/2) (16). None of these four countries have reported any data regarding negative effects on graft function in the recipients of the kidneys exposed to ICM. Estonia performs an unenhanced abdominal CT scan and abdominal US on all their donors. The idea behind this policy is that with an unenhanced abdominal CT the donor is being screened for any abnormalities or pathological findings (and if indicated, this is supplemented with an enhanced CT scan), while doppler ultrasound is used to assess renal vascularization.

Since the introduction of CT scans in the 1970s, it has become an important tool offering fast and reliable diagnosis of various diseases, which accelerated the application within a broad framework in daily medical practice (17–20). The technique of ICM was introduced even before the invention of the CT scanner, but the chemical properties changed through the years; high osmolarity contrast media were replaced, because of its nephrotoxic properties, by low osmolarity contrast and iso-osmolar contrast agents (21).

In donor assessment, the use of CT scans has several (potential) advantages, namely an accurate detection of malignancies and more accurate assessment of organ quality (i.e., liver steatosis, renal atrophy, severity of atherosclerosis, or pulmonary embolism) compared to conventional modalities. In 2019, Mensink et al. performed a retrospective study to assess the additional value of CT scans in donor screening and concluded that, if a CT scan was added to the screening protocol, at least 7 unnecessary procurements (0.5% of all procurements) could be prevented, over a 5 year period, due to the identification of malignancies (22).

Also, in detecting aberrant (vascular) anatomy, for example the kidney and the liver, CT scans will provide valuable information. Multiple renal arteries are not a rarity with a reported incidence of 24%–28% and their presence causes a higher risk of potential complications at procurement with subsequent graft loss or DGF (23–26). The incidence of variants in hepatic arteries is even higher and ranges from 25% to 45%, insufficiently recognized aberrant anatomy could increase the risk of surgical injury during procurement (27–29). In living donor liver and kidney transplantation CT-scans are already routinely performed and proven essential for measuring total and residual liver volume and assess the anatomy (30). These same advantages could be gained in deceased donors and improve transplant outcome and graft survival (30–33). In lung transplantation, matching of the donor lung and recipient thorax is important to prevent size mismatch. Performing a chest CT results in better prediction of the total lung capacity, which therefore benefits the optimal matching and preoperative planning (4, 34).

However, every advantage has its disadvantage. If more accurate imaging is applied, the risk of incidental findings increases, resulting in additional tests and thereby prolonging duration of donor assessment or even cessation of a donor procedure. The extent of this risk is currently unknown and must be weighed against the likelihood of malignancy transmission. On the other hand, not performing a CT scan because of the fear of finding anomalies of unknown significance and a chance of leading to cessation of the donor, means the physicians are taking a calculated risk for transplanting a malignancy. From an ethical perspective, this could raise the question of whether it is safe to transplant these organs.

Also, transporting a potential donor that might be hemodynamically unstable to the CT could also be a challenge. In case of a DCD II (unsuccessful resuscitation) and DCD IV (cardiac arrest in a patient who is brain dead), performing a CT scan is probably in most of the cases impossible.

A CT scans is associated with higher costs compared in comparison to CXR and abdominal US; a chest and abdominal CT scan in Netherlands cost approximately €400 together, while the costs of a CXR and abdominal US together are less than €150 (35). But despite the extra costs, it could be more cost effective by timely cessation of a donor procedure in case of malignancy. Yet this assumption should also be considered in future studies.

This study has a few limitations that need to be addressed. First, not all countries approached replied to our survey and the majority of the countries were from Europe. However, several large and influential transplant organizations did respond. The response rate was 67%, which is in accordance with the response rate in patient and health care professional surveys in surgery (the average response rate was 53%, SD 25%) (36). Since only the countries that replied to the survey could be included, a certain selection bias should be considered. The survey was created by the author itself and reviewed by several procuring surgeons, which could have led to missing questions. For example, the survey did not contain the option to fill out whether chest CT is performed with or without ICM. Nevertheless, none of the respondents commented chest CT was performed using ICM. To define the countries to be approached the IRODaT registry was used instead of the international figures from the Global Observatory on Donation and Transplantation WHO-ONT, since the author was familiar with the IRODaT Registry. After comparing the data from both databases, in 80% of the countries the number of deceased donors was the same in both databases. In 20% of the countries the numbers differed by only a few numbers.

In conclusion, this overview shows that policies regarding radiologic screening in deceased donor organ management are quite different between various countries and transplant organizations throughout the world, based on different views on (the safety of) organ transplantation. Future research should focus on interviewing specific transplant centers or Organ Procurement Organisations regarding their policies. This study shows there is a need to prospectively investigate the value of CT scans in deceased organ donation. In such a study, we would suggest the following outcome measurements; changes in acceptance of the grafts based on the diagnostic imaging, better matching of donor-recipient (size measure for long and/or liver transplantation) and the incidence of detecting malignancies before procurement. This type of research could contribute to making decisions on policy changes evidence-based and well considered.

## Data Availability

The raw data supporting the conclusion of this article will be made available by the authors, without undue reservation.
