# Supplementary material for: Radiological Screening Methods in Deceased Organ Donation: An Overview of Guidelines Worldwide
Source: Transpl Int. 2022 May 19;35:10289. doi: 10.3389/ti.2022.10289 (PMC9161442; doi:10.3389/ti.2022.10289)
Supplement: Supplementary file 1 [file DataSheet4.pdf]

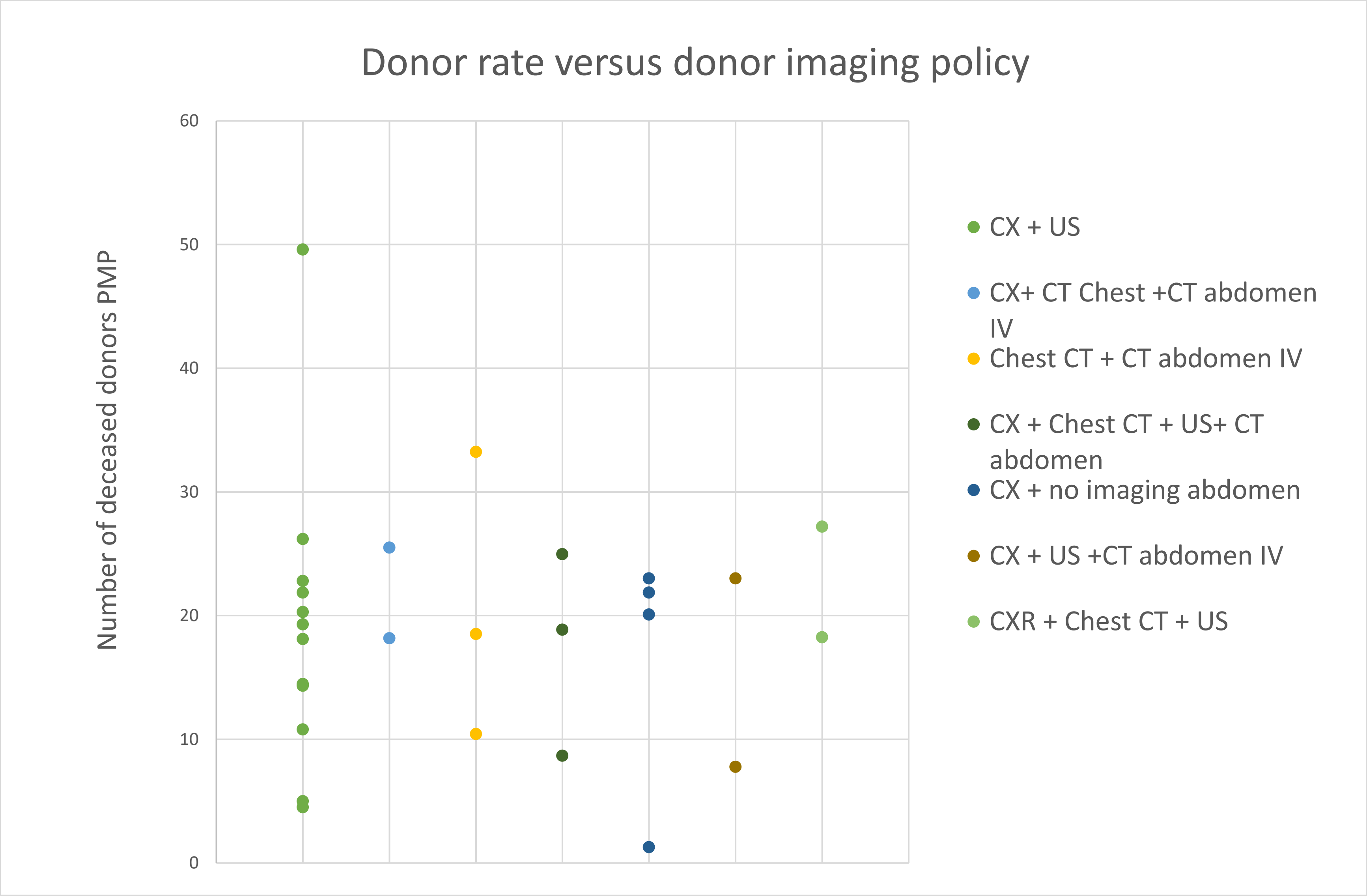

Supplementary Datasheet 4 I Graphical view of donor rate versus donor imaging policy
