# Supplementary material for: Radiological Screening Methods in Deceased Organ Donation: An Overview of Guidelines Worldwide
Source: Transpl Int. 2022 May 19;35:10289. doi: 10.3389/ti.2022.10289 (PMC9161442; doi:10.3389/ti.2022.10289)
Supplement: Supplementary file 2 [file DataSheet3.pdf]

Supplementary Datasheet 3 - Countries approached per region:

**Africa**	
Countries approached	1
Countries responded	1
Response rate	100%

**Asia**	
Countries approached	4
Countries responded	3
Response rate	75%

**Europe**	
Countries approached	22
Countries responded	16
Response rate	73%

**Middle America**	
Countries approached	4
Countries responded	1
Response rate	25%

**Middle east**	
Countries approached	3
Countries responded	2
Response rate	75%

**North America:**	
Countries approached	2
Countries responded	2
Response rate	100%

**Scandinavia:**	
Countries approached	4
Countries responded	4
Response rate	100%

**South America**	
Countries approached	6
Countries responded	2
Response rate	33%

**Oceania**	
Countries approached	2 (In one email, since it is one competent authority)
Countries responded	2 (In one reply, since it is one competent authority)
Response rate	100%
