# Supplementary material for: Radiological Screening Methods in Deceased Organ Donation: An Overview of Guidelines Worldwide
Source: Transpl Int. 2022 May 19;35:10289. doi: 10.3389/ti.2022.10289 (PMC9161442; doi:10.3389/ti.2022.10289)
Supplement: Supplementary file 3 [file DataSheet5.pdf]

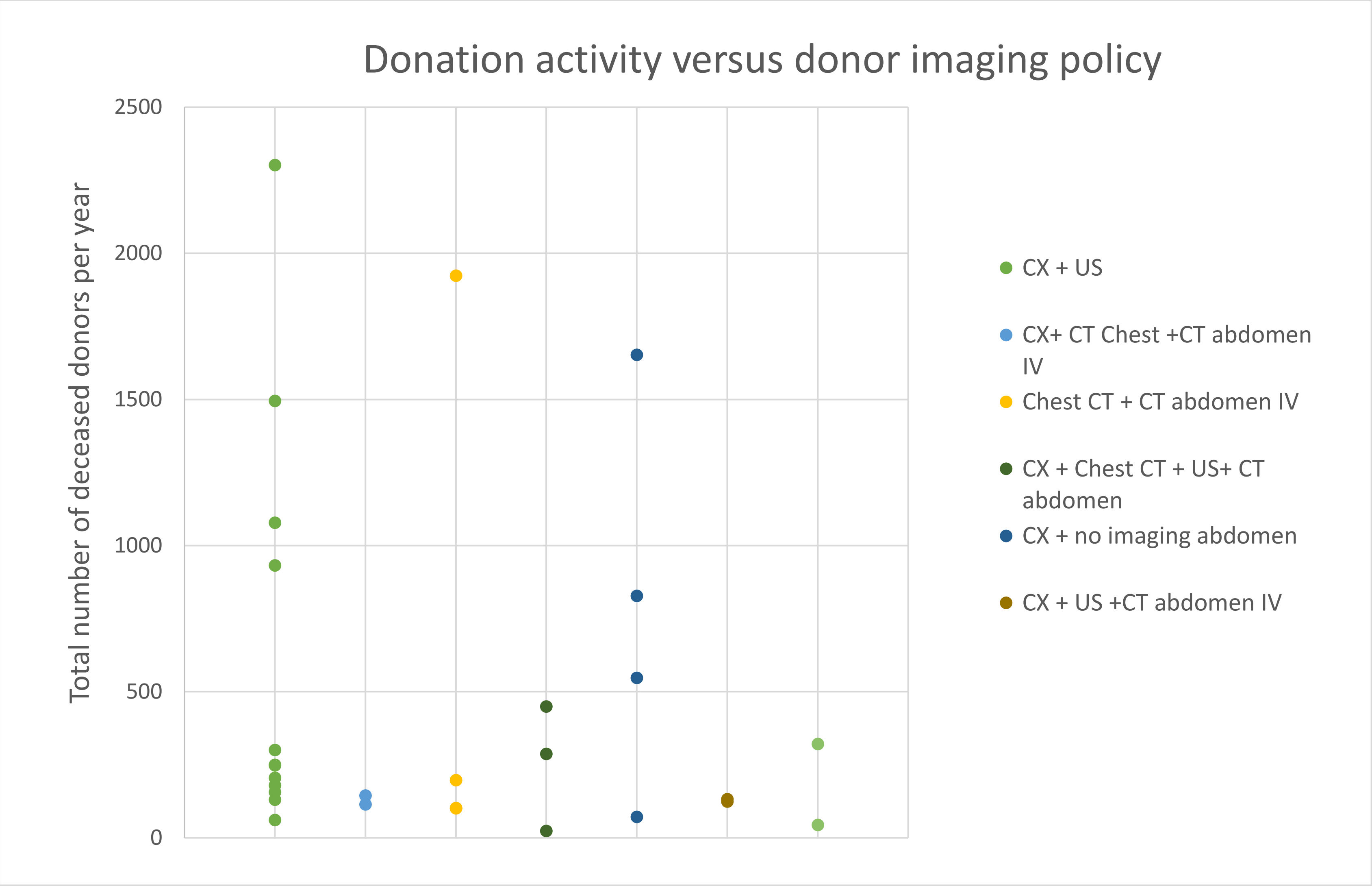

Supplementary Datasheet 5 I Graphical view of donation activity versus donor imaging policy

* In the above graphic the United States is not plotted. This country has a donation activity of 11.870 donors per year, since this value is an outliner, it took away the detail of the graphic and is therefore left out.
