# Supplementary material for: Radiological Screening Methods in Deceased Organ Donation: An Overview of Guidelines Worldwide
Source: Transpl Int. 2022 May 19;35:10289. doi: 10.3389/ti.2022.10289 (PMC9161442; doi:10.3389/ti.2022.10289)
Supplement: Supplementary file 4 [file DataSheet2.DOCX]

**Appendix 2**

**Questionnaire radiological screening in deceased donor organ donation**

1. We would like to publish the results of this questionnaire in a scientific paper. Do you have any objections against publication of this information, including mentioning your country/region?
   1. Yes
   2. No

**Procurement Abdominal Organs**

1. What is the preferred radiological screening method for the thorax in your region/country when ONLY abdominal organs are being procured?
   1. Chest X-ray
   2. CT Thorax
   3. Both
   4. Other:
2. What is the preferred radiological screening method for the abdomen when abdominal organs are being procured?
   1. Abdominal Ultrasound
   2. CT abdomen
      1. With contrast
      2. Without contrast
   3. Other:
   4. Both
3. Do you have any policies regarding incidental findings found on imaging of the abdomen?

Yes/no

Please comment – additional remarks

**Procurement Thoracic Organs**

1. What is the preferred screening method for the thorax in your region/country when thoracic organs are being procured?
   1. Chest X-ray
   2. CT Thorax
   3. Other:
   4. Both
2. What is the minimal radiological screening method for the abdomen when ONLY thoracic organs are being procured?
   1. Abdominal Ultrasound
   2. CT abdomen
      1. With contrast
      2. Without contrast
   3. Both
   4. Other:
3. Do you have any policies regarding incidental findings found on imaging of the thorax?

Yes/no

Explanation:

**General questions**

1. If a CT scan is not part of the standard screening protocol, in what percentage of potential donors has a CT thorax already been performed during hospital admission (before starting donor procedures?)

Number:

If you do not have actual percentages, please provide an estimated guess.

- 1. <10%
  2. 10-40%
  3. 40-60%
  4. >60%

1. In such cases, what was the reason to perform a CT thorax?
   1. Trauma screening
   2. Anomalies on the conventional imaging
   3. Other:
2. If a CT scan is not part of the standard screening protocol, in what percentage of potential donors has a CT abdomen already been performed during hospital admission (before starting donor procedures)?

Number:

If you do not have actual percentages, please provide an estimated guess.

- 1. <10%
  2. 10-40%
  3. 40-60%
  4. >60%

1. In such cases, what was the reason to perform a CT abdomen?
   1. Trauma screening
   2. Anomalies on the conventional imaging
   3. Other:
2. Do you have plans to change your policy in the near future?
   1. Yes
   2. No

Explanation:

1. Are there in your country/region any objections to use CT scans in the screening process of deceased donor organ donation?
   1. Yes (please check all options that apply)
      1. A CT scan may reveal anomalies of unknown significance, unintentionally leading to cessation of the donation procedure
      2. A CT with contrast may cause kidney injury
      3. A CT scan has no added value, above a X-thorax and abdominal ultrasound
      4. Performing a CT (with contrast) in a potential donor is regarded as a logistic challenge
      5. Other:
   2. No (please check all options that apply)
      1. It makes organ donation saver regarding detection of potential malignancies
      2. It provides extra information about the quality of the donor organ(s)
      3. It provides extra information about the anatomy of the donor organ(s).
      4. It provides extra information about the size of the donor organ(s).
      5. Other:

Explanation:

1. Are radiological donor images (CT, ultrasound or other) available to

- Retrieval surgeons before the donor operation

yes/no

- Accepting transplant surgeons during the allocation process

yes/no

- Accepting transplant physicians (pulmonologists, cardiologists, hepatologists, nephrologists)

yes/no

1. If applicable, please upload your protocol for radiological screening for deceased donor organ donation here
