# Supplementary material for: Radiological Screening Methods in Deceased Organ Donation: An Overview of Guidelines Worldwide
Source: Transpl Int. 2022 May 19;35:10289. doi: 10.3389/ti.2022.10289 (PMC9161442; doi:10.3389/ti.2022.10289)
Supplement: Supplementary file 5 [file DataSheet1.DOCX]

**Appendix 1:**

Search strategy used in PubMed:

(("cadaver*"[tw] OR "deceased"[tw] OR "post mortal"[tw] OR "post-mortal"[tw] OR "brain death"[tw] OR "coma"[tw] OR "comatose"[tw]) AND ("Tissue Donors"[Mesh] OR ("donor*"[tw] OR "donat*"[tw] OR "graft*"))) AND (“Tissue and Organ Harvesting”[Mesh] OR “Tissue and Organ Harvesting”[tw] OR “Organ Retrievals”[tw]) AND ("Diagnostic imaging"[Mesh] OR "Imaging, Diagnostic"[tw] OR "Medical imaging"[tw] OR "Imaging, Medical"[tw] OR "Radiography"[Mesh] OR "Radiography"[TW] OR "Diagnostic X-Ray" [TW] OR "Roentgenography" [TW] OR "Diagnostic X-Ray Radiology"[TW] OR "Diagnostic X Ray Radiology" [TW] OR "CT-scan"[tw] OR "CT-scans"[tw] OR "CAT-scan"[tw] OR "CAT-scans"[tw] OR "Computed Tomograph*"[tw] OR "Computer Tomograph*"[tw] OR "Computerized Tomograph*"[tw] OR "Computerised Tomograph*"[tw] OR "Computer Assisted Tomograph*"[tw] OR "CT angiography*"[tw] OR "CTA"[tw] OR "whole body CT"[tw])
